# Rapid and Accurate Varieties Classification of Different Crop Seeds Under Sample-Limited Condition Based on Hyperspectral Imaging and Deep Transfer Learning

**DOI:** 10.3389/fbioe.2021.696292

**Published:** 2021-07-23

**Authors:** Na Wu, Fei Liu, Fanjia Meng, Mu Li, Chu Zhang, Yong He

**Affiliations:** ^1^College of Biosystems Engineering and Food Science, Zhejiang University, Hangzhou, China; ^2^College of Information and Electrical Engineering, China Agricultural University, Beijing, China; ^3^Maize Research Institute, Jilin Academy of Agricultural Sciences, Gongzhuling, China; ^4^School of Information Engineering, Huzhou University, Huzhou, China

**Keywords:** crop seeds, hyperspectral imaging, classification model, spectroscopic analysis, deep learning

## Abstract

Rapid varieties classification of crop seeds is significant for breeders to screen out seeds with specific traits and market regulators to detect seed purity. However, collecting high-quality, large-scale samples takes high costs in some cases, making it difficult to build an accurate classification model. This study aimed to explore a rapid and accurate method for varieties classification of different crop seeds under the sample-limited condition based on hyperspectral imaging (HSI) and deep transfer learning. Three deep neural networks with typical structures were designed based on a sample-rich Pea dataset. Obtained the highest accuracy of 99.57%, VGG-MODEL was transferred to classify four target datasets (rice, oat, wheat, and cotton) with limited samples. Accuracies of the deep transferred model achieved 95, 99, 80.8, and 83.86% on the four datasets, respectively. Using training sets with different sizes, the deep transferred model could always obtain higher performance than other traditional methods. The visualization of the deep features and classification results confirmed the portability of the shared features of seed spectra, providing an interpreted method for rapid and accurate varieties classification of crop seeds. The overall results showed great superiority of HSI combined with deep transfer learning for seed detection under sample-limited condition. This study provided a new idea for facilitating a crop germplasm screening process under the scenario of sample scarcity and the detection of other qualities of crop seeds under sample-limited condition based on HSI.

## Introduction

High-quality seeds are conducive to continue excellent species and guarantee crop yield and quality. Due to the significant differences in climate, soil, and water resources in different regions, breeders have pointedly developed many crop varieties to adapt to the local planting environment. Growth rules, stress resistance, and biochemical characteristics of different varieties of crops vary greatly (Du et al., 2017; Zhang et al., 2020). For varieties that are still in the breeding stage, screening a variety with specific traits often needs to observe the phenotypic traits of the offspring plants, which is time-consuming and labor-intensive. As a seed carries all the genetic genes that develop into a plant, seed classification can be an alternative for screening variety with specific traits. For varieties that have been promoted widely, different varieties of seeds frequently circulate in the market, tending to be easily mixed, making the seed purity unable to be guaranteed. Conventionally, the manual vision inspection method based on the external phenotypic traits of seeds, like color, texture, and shape, is subjective and boring (Rashid and Singh, 2000). The more accurate methods based on the internal biochemical properties of seeds, such as DNA molecular markers (Ye et al., 2013) and protein electrophoresis techniques (Shuaib et al., 2007), rely on chemical agents and complex operation. Accordingly, it is necessary to develop a rapid and accurate method for the varieties classification of crop seeds.

As hyperspectral imaging (HSI) can obtain spectral and spatial location information simultaneously during one scan, it has the capability of probing the internal and external phenotypic traits of samples rapidly (Sendin et al., 2018). HSI has gained tremendous and continuous attention in breed screening (Feng et al., 2017), plant phenotyping (Qiu R. et al., 2018; Sun et al., 2019), and environment monitoring (Stuart et al., 2019). In seed-related tasks like determination of seed quality (Shrestha et al., 2016), diagnosis of seed diseases (Wu et al., 2020) and detection of seed components (Caporaso et al., 2018), HSI has been utilized as a rapid and accurate alternative. Since hyperspectral image contains a large amount of redundant collinear information, diverse linear and non-linear machine learning approaches, such as partial least squares discriminant analysis (PLS-DA), extreme learning machine (ELM), and least square support vector machines (LSSVM), were introduced to couple the relationship between seed spectra and a category label or component content (Caporaso et al., 2018; Kong et al., 2018; Weng et al., 2018).

In recent years, with the attention from academia and industry increasing, deep learning as the new state-of-the-art machine learning approach has also been applied in the spectral analysis field gradually (Jin et al., 2018; Wei et al., 2018; Yu et al., 2018). Compared with traditional approaches, deep learning can extract various low-level and high-level features automatically through a multilayered stack network structure (LeCun et al., 2015). This advantage can reduce the requirement of prior knowledge from specific tasks and human effort in feature engineering, which is very beneficial for analyzing redundant and high-dimensional spectral data.

However, typical deep learning models, such as deep networks, generally have serious big data dependencies. A high-performance deep network requires enough samples to adequately learn the feature patterns hidden in the massive and redundant spectral data. Unfortunately, in some tasks like seeds screening with specific traits during the breeding process or quality detection of precious agricultural products, it is challenging to establish a large-scale, high-quality dataset because of the high cost of obtaining and labeling samples (Lee et al., 2016; Xu et al., 2017; Sun et al., 2019). Besides, the precious data acquired at great expense is straightforward to be outdated and difficult to be reused in new tasks, which dramatically limits the rapid application of well-performing methods like the deep network in spectral analysis. Another problem is that the deep networks developed for different tasks are generally based on a common assumption, that is, training and testing data lie in the same feature space and have the same distribution (Weiss et al., 2016). Therefore, even for similar tasks, the tiny differences in the distribution of different datasets will make the network not reusable.

The emergence of transfer learning brings hope for solving the above two problems. The transfer learning method allows the training and testing data to lie in different feature spaces. It mainly investigates how to transfer useful knowledge from the relevant source domain to the target domain (Pan and Yang, 2010). This property not only relieves the demand for a large number of samples in the target task but makes reusing the shared knowledge like model structure and feature representation in the source domain possible. The target task can be expected completed, using limited samples and computation overhead. Deep transfer learning is the product of the combination of deep learning and transfer learning. It aims to study how to use the deep neural network to transfer knowledge and has been widely used in the computer vision field (Mohanty et al., 2016; Ghazi et al., 2017; Tan et al., 2018).

However, the deep transfer learning technique has not received much attention in the field of spectral analysis. Most studies perform task analysis at a pixel level based on remote sensing images, such as poverty mapping (Xie et al., 2016), image superresolution processing (Yuan et al., 2017), and crop yield prediction (Wang et al., 2018). For ground spectral images, Liu et al. (2018) showed the effectiveness of deep transfer learning in predicting soil clay content in different soils. For seeds of different crops, there are also certain similarities, for example, the structure of the seeds. Most seeds contain a seed coat, an embryo, and endosperm. These parts contain some common chemical components, like starch, fat, and enzymes, which are necessary for a seed to develop into a seedling (Beníteza et al., 2013; Zhao et al., 2018). This commonality may lead to the similarities among the spectral characteristics of different crop seeds. Therefore, when constructing a deep model for seed varieties classification of a specific crop based on HSI, the knowledge in the model is possible to be transferred to the classification tasks of other crop seeds. In this study, we aimed to investigate the feasibility of the deep transfer learning technique for the varieties classification of different crop seeds based on HSI.

The specific objectives were: (1) to develop a deep network model with excellent performance based on a sample-rich dataset; (2) to transfer common knowledge to the varieties classification of other crop seeds with sample-limited datasets through the deep network; (3) to evaluate the impact of training set size on the performance of transfer learning; and (4) to visualize the transferring process of deep network and the classification results. We hope to provide a common framework for rapid and accurate varieties classification of crop seeds under sample-limited condition through this study.

## Materials and Methods

### Sample Collection and Dataset Description

This study investigated five kinds of crop seeds, including pea, rice, oat, wheat, and cotton. All images were obtained by the same line-scanning near-infrared HSI system, covering a spectral range from 874.41 to 1,733.91 nm with a resolution of 5 nm (Wu et al., 2018). An ImSpector N17E imaging spectrograph (Spectral Imaging Ltd., Oulu, Finland) and a Xeva 922 CCD camera (Xenics Infrared Solutions, Leuven, Belgium), configured with an OLES22 lens (Spectral Imaging Ltd., Oulu, Finland), were the critical components of this system. In addition, the illumination was provided by two 150 W tungsten halogen lamps (3900e Lightsource; Illumination Technologies Inc.; West Elbridge, NY, United States) set symmetrically under the camera. Multiple seed samples placed on a dark plate flowed a miniature conveyer belt to achieve batch detection. A hyperspectral image, containing 256 spectral channels, could be obtained through each scan by this system and then calibrated using the following formula.

$$I_{c}=\frac{I_{r}-I_{d}}{I_{w}-I_{d}}$$

where *I*_*r*_ and *I*_*c*_ represented the raw hyperspectral image and the corrected image, *I*_*w*_ and *I*_*d*_ represented the white and dark reference image. Each seed in the hyperspectral image was regarded as a region of interest (ROI). To get the mask of all the ROIs, simple threshold segmentation and morphological operation were performed on the channel image with the strongest contrast between the background and the seeds. Then the spectral vectors of all pixels within each ROI were extracted, and the bands in head and end ranges were removed to avoid noise introduced by the instability of the system. The reserved spectra with a range of 975–1,646 nm were further processed by wavelet transform (WT). The spectrum vector, representing a seed sample, was finally obtained by averaging all the transformed pixel spectra in one ROI.

Five spectra datasets with similar but different distributions were established in this study. Their detailed collection parameters and description information were summarized in Table 1. It should be noted that different parameters were set for imaging different crop seeds clearly since different seeds have different external phenotypes, such as size, height, and color. The most abundant dataset, the Pea dataset, contained a total of 10,420 samples from four varieties named Baiyan (2697), Heiyan (2,848), Changshouren (2,849), and Zhewan 1 (2,026), which were widely cultivated in southern China. Peas of the first two varieties generally need to be roasted before eating, while the latter two can be directly eaten due to the high water and sugar content. All the seeds were purchased from the Lvfeng seed company in Hangzhou, Zhejiang, China, in 2018. The dataset corresponding to each variety was randomly divided into a training set, a validation set, and a testing set at a ratio of 3:1:1. Then those independent subsets with the same category were merged and shuffled. Because of its large volume of data, the Pea dataset was selected as the source dataset.

**TABLE 1:** Description of the source and target datasets.

Datasets		Parameters^1^	#Variety	#Total	#Training	#Validation	#Testing
Source	Pea	(15.5, 3, 12)	4	10,420	6,252	2,084	2,084
Target	Rice	(9, 3, 11)	3	750	150	300	300
	Oat	(15.2, 3, 11.5)	4	1,000	200	400	400
	Wheat	(15, 3, 13)	5	1,250	250	500	500
	Cotton	(14, 3, 11.5)	7	1,750	350	700	700

*Parameters^1^ represents parameters of the hyperspectral imaging system, including the distance between the camera and the seed plate (cm), the exposure time of the camera (ms), and the speed along the x-axis of seeds movement (mm.s^–1^).*

The other four sample-limited datasets were used as the target datasets designed to contain different numbers of seed varieties for investigating their impact on the transferring effect. Each variety in these datasets contained 250 samples and was further divided into three subsets at a ratio of 1:2:2 to reflect sample-limited condition.

The first target dataset consisted of 750 spectral samples of three varieties of rice seeds, including Yongyou 9, Nuoyou 6211, and Zhongbaiyouhuazhan. These varieties are all hybrid rice with indica property and belong to hybrid indica-japonica, hybrid indica-glutinous, and hybrid indica rice, respectively. All seeds were collected by the College of Agriculture and Biotechnology, Zhejiang University in 2019.

The second dataset was the Oat dataset with the same number of varieties as the source dataset. It contained 1,000 samples from four varieties named Bayan 6, Dingyan 2, Muwang, and Jizhangyan 4, which were widely planted in the grasslands of northern China. The seeds harvested in 2017 were kindly provided by the Academy of Agricultural and Animal Sciences, Inner Mongolia, China.

A total of 1,250 samples from five varieties of wheat seeds, including Zhenmai 9, Annong 1,124, Longpingmian 6, Shannong 102, and Weilong 169, formed the Wheat dataset. These five varieties were extensively cultivated in the winter wheat regions of southern China. The seed samples were friendly provided by Anhui longping high-tech seed industry Co., Ltd., in Hefei, Anhui, China, in 2018.

The fourth dataset, the Cotton dataset, was consisted of 1,750 samples of seven varieties of cotton seeds. They were Jinxin 5, Jinxin 7, Shennongmian 1, Xinjiangzaomian 1, Xinluzaomian 29, Xinluzhong 52, and Xinluzhong 42. These varieties were mainly grown in Xinjiang Uyghur Autonomous Region, the largest cotton-producing region in China. And the cotton seeds were collected by Shihezi University in 2016.

In this study, multiple deep neural networks with different structures were first developed, using the source dataset. Then the optimal deep model was selected as the model to be transferred by comparing the classification accuracies. The transfer learning technique was investigated to transfer useful knowledge from the optimal deep model to the analysis of four target datasets. The training set of each target dataset was further transformed into 10 datasets to analyze the impact of sample size on transfer learning by randomly selected 10–100% samples from the original training set. Four commonly used multivariate analysis methods, including two linear methods: linear discriminant analysis (LDA) and PLS-DA, and two non-linear methods: multilayer perceptron (MLP) and support vector machines (SVM), were introduced as the benchmarks.

### Deep Classification Models Development

In the computer vision field, the huge image library, ImageNet, has spawned many excellent deep learning models like VggNet, InceptionNet, and ResNet (Krizhevsky et al., 2012). The specialness of VggNet is using small convolution kernels. The designers believed that using multiple convolution layers equipped with a 3 × 3 kernel to replace a convolution layer with a 5 × 5 kernel could reduce the network parameters and increase non-linear mapping, thereby increasing the representation capability (Simonyan and Zisserman, 2015). ResNet is also an outstanding network with many variations. What makes it unique is the introduction of residual learning. The residual module directly bypasses the input of a particular layer to the output, which makes ResNet only need to learn the residual between the input and the output (He et al., 2016). This manner solves the problem of performance degradation when the network depth increases. InceptionNet was born in the ILSVRC2014 competition. The most significant innovation of this network is introducing a module called “Inception” to replace the typical structure of the convolution layer, cascading the pooling layer (Szegedy et al., 2015). This Inception module contains four branches with different receptive fields to perceive the input patterns. By utilizing this module, InceptionNet can increase its width and learn more local features of different scales.

**FIGURE 1:**
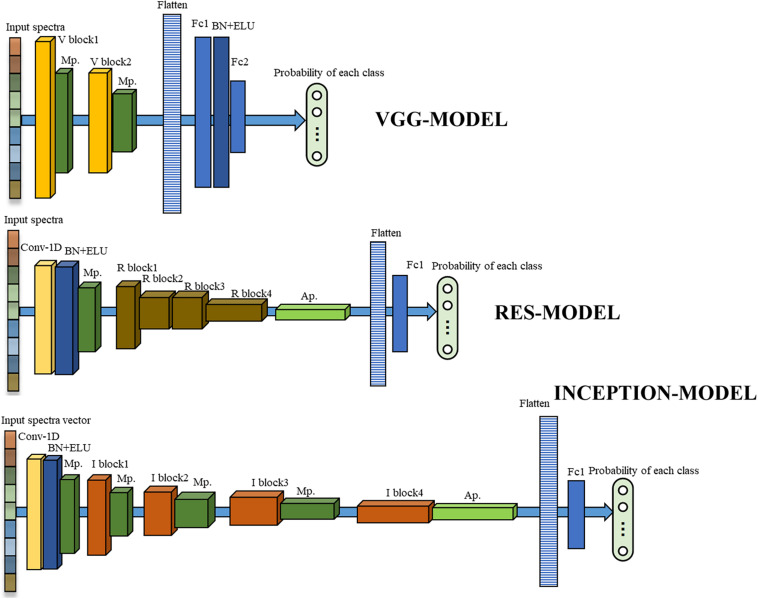
The structures of three developed deep neural networks. The orange, brown, and red cubes represented the V block, R block, and I block in Figure 2, respectively. The dark green and light green cubes represented the max-pooling layer and the average pooling layer, respectively. The yellow and blue cubes represented a one-dimensional convolutional layer, BN cascade activation function ELU, respectively. The striped bars represent flattened layers, and the light blue bars represented fully connected layers. The length, width, and height of the cubes and the bars in the Figure were drawn according to the dimensional size of data in each layer for a more intuitive display.

Inspired by these network structures, three one-dimensional deep neural networks were developed for the source dataset in this study, as shown in Figure 1.

**FIGURE 2:**
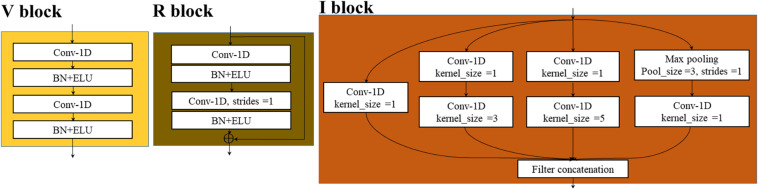
The inner structures of three typical blocks.

The first one was VGG-MODEL. Two V blocks (Figure 2), containing two convolution layers equipped with a 1 × 3 kernel were designed to extract the feature patterns hidden in the spectral vectors. A batch normalization (BN) and an activation function, exponential linear unit (ELU), were inserted after each convolution to reduce the overfitting risk and speed up the convergence process. The number of convolution filters was set to 16 for the first V block and 32 for the second V block. A max-pooling layer was placed behind each V block to reduce the feature dimension. A flatten layer was set after the last max-pooling layer to convert its output feature into a one-dimensional vector form. Layer Fc1 and Fc2, consisting of 64 and 4 neurons, were used to perform the classification task like traditional neural networks. BN and ELU were also used behind Fc1. VGG-MODEL finally output the probability of the input spectral vector belonging to each category through a softmax function.

The second one was RES-MODEL. The first part of this network was similar to half of the V block, which contained a convolutional layer followed by BN, ELU, and a max-pooling layer. The difference was that the convolutional layer used 32 kernels, with a size of 1 × 7. The second part consisted of four cascaded residual modules, R block. This module was similar to the V block but added a transmission channel from input to output (Figure 2). The number of convolutional filters in the first R block was 32 and was doubled as the blocks going deeper. An average pooling layer was placed after the last R block to average the features in the spectral dimension. This layer could decrease the parameters in fully connected layers, thereby reducing the overfitting risk. The last part of RES-MODELDE was similar to that of VGG-MODEL but was equipped with one fully connected layer, Fc1, with four neurons.

The third one was INCEPTION-MODEL. Having the same structure as that of RES-MODEL, the first part of this network utilized 16 convolution filters, with a size of 1 × 3. It was followed by four I blocks (Figure 2), each of which cascaded a max-pooling layer except the last one. The number of filters in the first I block was 16 and was doubled as the blocks going deeper. As shown in Figure 2, the I block transmitted its input to four parallel branches. Three of them were convolution layers with 1 × 1, 1 × 3, and 1 × 5 kernels, respectively. They were employed to extract local spectral features at different scales. A 1 × 1 convolution was placed before 1 × 3 and 1 × 5 convolution to reduce the number of input channels. The last branch performed the max-pooling operation. The end of the INCEPTION-MODEL was similar to that of RES-MODEL.

To fairly compare the performance, these three deep networks employed cross-entropy as the objective function and used stochastic gradient descent (SGD) optimization algorithm. The learning rate and momentum were all set to 0.001 and 0.9, respectively. After debugging many times, the number of samples input into the network at one time, *batch_size*, was set to 128, and the number of training iterations, *epoch*, was set to 400. All networks were trained, using the training set of the source dataset. The model for each network that obtained the highest accuracy on the validation set was saved. The effectiveness of the model was evaluated on the testing set. The detailed parameters of these three networks were shown in Supplementary Table 1.

### Transfer Learning Strategy

**FIGURE 3:**
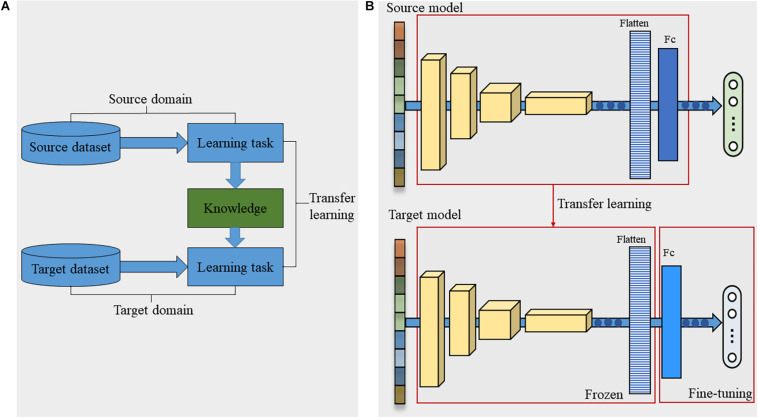
Transfer learning strategy. **(A)** The typical process of transfer learning. **(B)** The deep transfer learning strategy in this study. The yellow cubes of different sizes represented multiple cascaded convolution layers. The striped bars and blue bars still represented the flattened layer and the fully connected layer.

As an emerging tool in machine learning, transfer learning was proposed to remit the requirement of models for sufficient training data by transferring available knowledge from the relevant source domain to the target domain (Pan and Yang, 2010). The typical process of transfer learning was shown in Figure 3A. We defined a domain 𝒟 = {𝒳,P(𝒳)} where 𝒳 represented a feature space and P(𝒳) represented its probability distribution, and a task 𝒯 = {*y*,*f*(.)}where *y* represented a label space and *f* represented a transformed function. When the task 𝒯 was performed in the domain 𝒟, *f* modeled P(*y*| *x*), where *y* ∈𝒴, *x* ∈𝒳. In the transfer learning field, there are two domains: source domain 𝒟_*S*_ with task 𝒯_*S*_ and target domain 𝒟_*T*_ with task 𝒯_*T*_. The main goal of transfer learning is to improve the performance of the transformed function in the target domain *f*_*T*_(.), using the knowledge learned in 𝒟_*S*_ and 𝒯_*S*_, where 𝒟_*S*_ (or 𝒯_*S*_) and 𝒟_*T*_ (or 𝒯_*T*_) are different but relevant.

For deep transfer learning, *f*(.) is various deep models designed for specific tasks. These deep models contain rich knowledge. Some knowledge is closely related to the specific task, while others can be shared between different tasks or objects. Deep transfer learning aims to transfer the common knowledge into the current target task to avoid learning this knowledge repeatedly, thus achieving rapid modeling. The structure of the model and the weight of the network are two important types of knowledge contained in deep models. In this study, the structure of the optimal deep model based on the source dataset was reused to simplify and shorten the modeling process. Since the number of seed varieties varies with different crops, the number of neurons in the output layer of the model was modified correspondingly. As the initial weights greatly influence the convergence speed and the final performance of the model, this study transferred the weights of the optimal deep model based on the source dataset to the models based on the target datasets according to the network structure. Since the number of output neurons in the deep models based on the Rice, Wheat, and Cotton dataset differed from that in the source model, the weights of the last fully connected layer in these models needed to be randomly initialized.

During the transferring process, the weights of the layers before the flatten layer were frozen, and the target datasets were used to fine-tune the subsequent fully connected layers (Figure 3B). The first reason was that the target dataset was too small to retrain the entire network. The second reason was that the convolutional layers before the flatten layer might have extracted important feature patterns of the seed spectra, which could be reused in the target tasks. According to the size of the target datasets, the *batch_size* of the transferred network was set to 3, and the learning rate was set to 0.0001. The other configurations were the same as the source model.

### Comparison Methods

In this study, the deep neural networks based on the source dataset and four target datasets were compared with conventional linear and non-linear multivariate analysis methods to confirm their validities in spectra analysis from both data-rich and data-poor sides.

LDA aims to find an optimal projected direction for raw variables. In the projected feature space, samples between classes hold maximal dispersion, while samples within classes hold minimal dispersion (Gerhardt et al., 2019). This projection manner facilitates transforming the samples into a linear separable state. The number of variables in the projected space, *n_lda*, is the only parameter that needs to be adjusted. We set *n_lda* to 1–20 and selected the optimal *n_lda* according to the classification performance of LDA.

The core principle of PLS-DA is also to conduct a linear transformation. Unlike LDA, the transformed latent variables (LVs) can carry the primary information hidden in the raw variables and maximize the correlation between the independent and the dependent variables (Kandpal et al., 2016). In spectral analysis, the number of LVs, *n_pls-da*, that minimize the sum of predicted residual error was usually selected. The range of *n_pls-da* was also set to 1∼20 in this study.

SVM can enable raw linear inseparable variables to transform into a linear separable space through a non-linear kernel function (Gerhardt et al., 2019). Radial basis function (RBF) kernel was often used with SVM in many spectral analysis tasks because of its ability to cluster samples with the same categories closely and make them linearly separable. In this study, SVM equipped with RBF kernel was introduced as a non-linear benchmark. Two parameters, penalty coefficient *c* and kernel parameter *g*, were set to {10, 100, 1,000, and 10,000} and {0.1, 0.01, 0.001, and 0.0001}, respectively.

MLP is a fully connected artificial neural network with one or more hidden layers (Taud and Mas, 2017). To obtain the optimal performance, a total of 32 structures were attempted to process the source dataset, which contained one to four hidden layers, and each was equipped with eight configurations for nodes in hidden layers. The number of nodes in hidden layers of the structure with four hidden layers was set to [(200-100-50-25), (180-90-45-23), (160-80-40-20), (140-70-35-18), (120-60-30-15), (100-50-25-13), (80-40-20-10), (60-30-15-8)], and was simplified as the number of the hidden layer decreases. For the target datasets, 24 same structures with one to three hidden layers were tried to get the optimal classifier.

In addition, to further verify the role of the shared features for effective transfer learning, a hyperspectral dataset in the remote sensing field, Indian-Pines^1^ was introduced. It is a 145 × 145 × 224 cube, containing 10,249 effective pixels of 16 categories, whose size was similar to that of the Pea dataset. These pixels were also randomly divided into a training set, a validation set, and a testing set at a ratio of 3:1:1. The number of the bands for analysis was reduced to 200 by removing the bands absorbed by water. To eliminate the influence of factors, such as the deep model, the structure of the optimal deep model based on the source Pea dataset was used to train the Indian-Pines dataset and was recorded as Model 0. Then, Model 0 was transferred to the other four target datasets.

The parameters of all models in this study were adjusted toward the optimal states, using the corresponding validation set. All models were coded, using python language in Spyder 3.2.6 environment (Anaconda, Austin, TX, United States). The famous machine learning library, Sklearn^2^, was introduced to implement the conventional models, and the popular deep learning framework, Keras, was employed to program deep models. A Win10 64-bit operating system with Inter (R) Core (TM) i5-8500 CPU and 8 GB RAM constituted the primary platform.

### Model Visualization

Model visualization is significant for intuitively understanding the decision-making mechanism and clearly showing the computational result. In this study, visualization techniques were investigated from the perspective of the training process of the deep model and the classification results of the crop seeds. The raw seed spectra of different datasets and the feature representation of different layers in the optimal deep models based on the source Pea dataset and the deep model based on the Indian-Pines dataset were extracted. Their distributions were then expressed by t-distribution stochastic neighbor embedding (t-SNE). As an effective method for high-dimensional data visualization, t-SNE converts the similarity between sample points in high-dimensional space into Gaussian joint probability form and constructs a similar probability distribution in low-dimensional space (van der Maaten and Hinton, 2008). The ability to maintain the local structure of data is conducive to observing data patterns in low-dimensional space. Moreover, the advantage of HSI to obtain both spatial and spectral information was fully exploited in this study. The label of the sample predicted by the deep model was projected into the corresponding spatial position and represented by different colors to establish classification maps of crop seeds.

## Results and Discussion

### Spectroscopic Analysis

**FIGURE 4:**
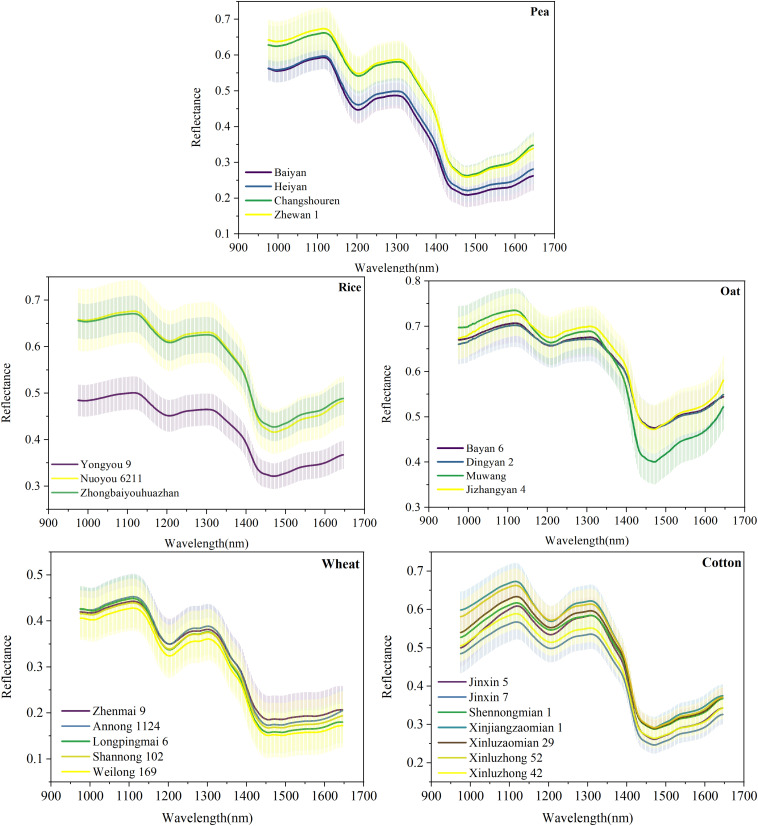
The average spectra with the standard deviation of five crop seeds.

The average spectra with the standard deviation of different varieties of seeds in five datasets were shown in Figure 4. Obviously, these spectral curves possessed similar fluctuation patterns and locations of peaks and valleys. The absorption bands at approximately 1,119.45 and 1,206.92 nm were caused by the second overtone of carbohydrates (C–H stretch) (Wu et al., 2019). The peak near 1,307.97 nm (in the range of 1,254 –1,348 nm) was reported to be associated with the combinations of the first overtone of amide B (N–H stretch) and the fundamental vibrations of amide II and III (C–N stretch and N–H in-plane bend) (Daszykowski et al., 2008). The band at 1,469.95 nm (in the region of 1,410–1,502 nm) could be attributed to the first overtone of Amide A (N–H stretch), which might be the critical band for protein detection (Daszykowski et al., 2008; Ribeiro et al., 2011). The similar chemical components in different crop seeds led to the similarities between the spectral curves. This meant that certain shared features might be hidden in the spectral information of different crop seeds, which provided the possibility for effective transfer learning.

However, for different varieties of seeds of the same crop, some heterogeneities also existed between their spectral curves due to the content difference of chemical components. For example, the spectral curves of four varieties of pea seeds were naturally divided into two groups. Baiyan and Heiyan formed one group, while Changshouren and Zhewan 1 formed the other one. This trend was consistent with the classification results according to the edible manner resulted from the content difference of sugar and water. In addition, for the Rice dataset, it was because of the introduction of the japonica characteristic that the reflectance of variety Yongyou 9 was quite different from the other two varieties. Qiu Z. et al. (2018) also confirmed spectra differences existed between different varieties of rice seeds. Nie et al. (2019) found the optical characteristics of different varieties of hybrid okra and luffa seeds were very different. The metabolic analysis results showed that the content of components of different seeds varied greatly. The heterogeneity of the spectral features between different varieties laid the basis for using HSI to classify different varieties of crop seeds.

### Classification Results on Source Dataset

The accuracies and the optimal parameters of all models on the training set and the testing set of the source dataset were summarized in Table 2. The overfitting phenomenon for all models was not serious due to the large-scale training set that might contain the spectral patterns in the testing set.

**TABLE 2:** The classification accuracies and optimal parameters of all the models on the source dataset.

Methods	Parameters^2^	Training (%)	Testing (%)
VGG-MODEL	(16, 32, 128, 201)	99.98	99.57
RES-MODEL	(32, 32, 64, 64, 128, 194)	99.76	99.14
INCEPTION-MODEL	(16, 32, 64, 128, 256, 349)	100	99.09
LDA	(1)	99.39	98.90
PLS-DA	(20)	87.14	86.90
SVM	(10^4^, 10^–3^)	99.70	99.28
MLP	(200, 100, 50, 25)	93.81	93.52

*Parameters^2^ represents (number of major convolution filters, best epoch) for deep models, (n_lda) for LDA, (n_pls-da) for PLS-DA, (c,g) for SVM, (number of nodes in hidden layers) for MLP.*

The accuracies of three deep models on the testing set were all above 99%, which was higher than most conventional methods. Owing to the convolution operation, the deep models could extract much discriminative information hidden in the raw redundant spectral data. Their performance superiorities were predictable. VGG-MODEL, with an accuracy of 99.57% on the testing set, was slightly conspicuous than the other two models. Generally speaking, the difficulty of improving performance increases with the performance of the model being better. For example, in the 2014 ILSVRC competition, a 22-layer InceptionNet won the championship with a top-five error rate of 6.7% that was only 0.6% lower than the runner-up, VGGNet, with a 19-layer structure (Szegedy et al., 2015). In addition, the high version of Inception, Inception-v4, achieved a top-five error rate of 3.08% that was only 0.42% lower than the previous version, Inception V3 (Szegedy et al., 2016).

Since the structures of the three deep models were continuously adjusted to the optimal states according to the source dataset, they possessed different depths. In this study, INCEPTION-MODEL and RES-MODEL had a deeper structure than VGG-MODEL. In general, the deeper the model is, the richer the extracted features are. But this was based on an enough big dataset like ImageNet, and it should be guaranteed that the gradient would not disappear during model training. Zhang et al. (2019) developed a network with an inception structure that showed better performance than a comparison network, Model 3, with a similar structure to VGG-MODEL. However, the authors also pointed out that the superiority of the deep model was not in processing small datasets. In their study, Model 3 could not learn enough effective patterns from a few samples. The authors also indicated that the performance of Model 3 improved significantly when the size of the dataset increased slightly. The source dataset in this study was much larger than all the datasets in their study and was enough for VGG-MODEL training. For structures like ResNet, Zhu et al. (2019) compared the performance of a developed ResNet with a general deep convolutional neural network on a cotton dataset. Also, they found that ResNet was not as effective as the latter one.

The structure of the optimal model for a specific dataset was the result of a constant tradeoff and adjustment. It was closely related to the size and distribution of the sample set. A complex deep network could not always obtain higher performance than a simple one. In this study, for the source dataset, VGG-MODEL with the simplest structure and the shallowest depth won a small victory when faced with the relatively complex INCEPTION-MODEL and RES-MODEL. For conventional models, the accuracies of different methods on the testing set varied greatly. SVM performed best, followed by LDA. Thus, if we use traditional multivariate analysis methods, many models need to be tried and compared to determine the optimal one (Liu et al., 2017; Bao et al., 2019; Nie et al., 2019). Conversely, deep models will generally achieve satisfactory results if the training data are sufficient and the structure is designed reasonably. In the field of spectral analysis, deep learning is a very competitive and potential tool.

### Classification Results on Target Datasets

**FIGURE 5:**
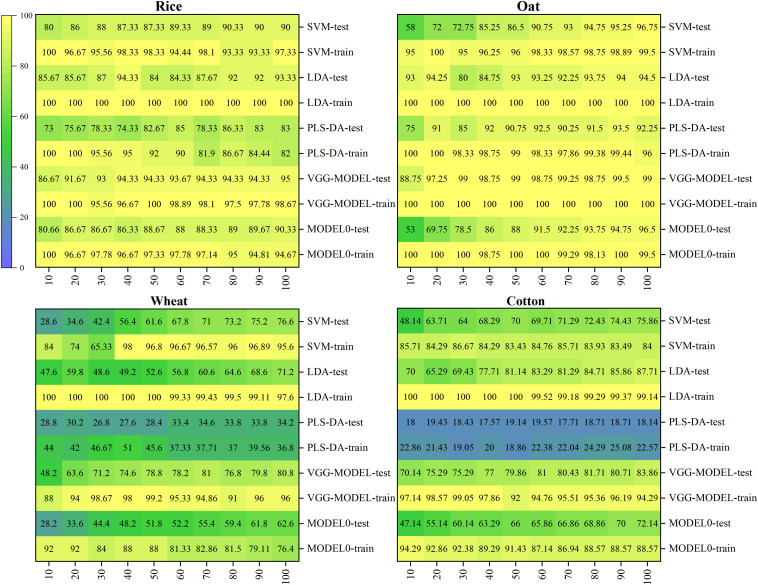
The classification accuracies of all the models on the four target datasets.

Although the deep network might not perform well on a small dataset, its advantages would carry forward again after combining with transfer learning. To verify the effect of deep transfer learning, the slightly better-performing VGG-MODEL was used as the source model to be transferred in this study. Ten training sets with different sizes were built based on the original training sets to investigate the influence of training set size on the transferring effect. The classification results of the deep transferred model and the comparison methods were shown in Figure 5.

It could be seen that the deep transferred model was the only model that consistently performed well on the four datasets. For the 100% training set that was still very small compared with the training set of the source dataset, the deep model achieved accuracies of 95, 99, 80.8, and 83.86% on the testing sets of Rice, Oat, Wheat, and Cotton datasets, respectively. It was because of combination with transfer learning that the deep learning model could also obtain satisfactory results on these datasets. Transfer learning enabled deep learning to take advantage of itself and avoided the requirement for a mass of samples (Tan et al., 2018). As similar patterns existed among the spectra vectors of different crop seeds, varieties classification of different crop seeds belonged to different but similar tasks in the same domain. Thus, transfer learning was very suitable for varieties classification of different crop seeds in this study.

However, if the difference between the target dataset and the original dataset was too large, it might cause a negative transfer. In this study, Model 0, the deep model based on the Indian-Pines dataset, achieved accuracies of 98.31 and 90.05% on the training set and the testing set, respectively. But its performance was worse than the deep transferred model based on the source Pea dataset and most conventional multivariate analysis methods when transferred to the four target datasets (shown in Figure 5). This poor performance could be expected since the Indian-Pines dataset and the seed datasets in this study were quite different in sampling scenarios, spectral resolution, and spectral modes. This result illustrated the importance of the similarity between the features of the source dataset and the target dataset for effective transfer learning in this study. When there is a vast difference between these two datasets, the direct transfer may lead to undesirable results. More effective transfer learning methods need to be studied in the future.

For other conventional models, although they could also achieve good performance on some datasets, they could not always perform well on all. For example, for the 100% training set, LDA achieved an accuracy of 87.71% on the Cotton dataset, which was even higher than that achieved by the deep transferred model. However, it just obtained accuracies of 93.33, 94.5, and 71.2% on the Rice, Oat, and Wheat datasets. SVM performed relatively stable, just like previous research (Qiu Z. et al., 2018; Bao et al., 2019; Nie et al., 2019). It achieved accuracies of 90, 96.75, 76.6, and 75.86% on the four datasets when using the 100% training sets. As expected, MLP performed much worse than the deep neural network. For the Wheat dataset, it only got an accuracy of 32.4% on the testing set, which was just a little better than random guessing. The shallow neural network could not extract valuable discriminative information from redundant spectral data, which led to unsatisfactory results (Chen et al., 2014). PLS-DA, commonly used in spectral analysis, was also very unstable. Although it could obtain an accuracy of 92.25% on the Oat dataset, it performed rather severely on the Wheat and Cotton datasets, which contained more varieties. Its performance was consistent with the results of Nie et al. (2019). With the increase of the number of seed varieties, the possibility of samples being linearly separable became smaller, and the difficulty of distinguishing different varieties became greater. In a word, for traditional multivariate analysis methods, different datasets might correspond to different optimal models. Conversely, the deep transferred models based on the source Pea dataset could generally achieve satisfactory results.

In addition, it was worth mentioning that the deep transferred model based on the source dataset could also achieve good results when fine-tuned, using tiny datasets. For example, when using the 10% training set, which only contained five samples for each variety, it could achieve accuracies of 86.67, 88.74, and 70.14% on the Rice, Oat, and Co tton datasets. And the accuracy rose rapidly with the increase of training set size. Even on the Wheat dataset, where all models failed, the deep transferred model outperformed all the conventional methods. Deep transfer learning brings hope for scenarios with very limited samples. On the contrary, the accuracies of most conventional methods were very low when trained, using such a small dataset. The result that LDA got a high classification accuracy of 93% on the Oat dataset was unexpected. The reason might be that this small training set just fitted the classification rule of LDA because its accuracy dropped to 80% soon for the 30% training set and then slowly increased.

Moreover, it could be observed that almost all the models showed high accuracies on the Rice and Oat datasets but performed poorly on the Wheat and Cotton datasets. The sample distribution of a dataset with few categories was generally simple. Contrarily, the distribution of a dataset with more categories was relatively complicated, which was not conducive to classification. Thus, the dataset was an essential factor affecting the performance of models (Özdemir et al., 2019; Zhang et al., 2019). In addition, it could be seen that the deep transferred model based on the source Pea dataset got the best performance on the Oat dataset. Using the 20% training set, it obtained an accuracy of 97.25% on the testing set. Since the Oat dataset had the same number of varieties as the source dataset, all the weight parameters in the source model, including the weights in the last fully connected layers, could be transferred. This specialness allowed the maximum transferring of features in the source model.

### Model Visualization

**FIGURE 6:**
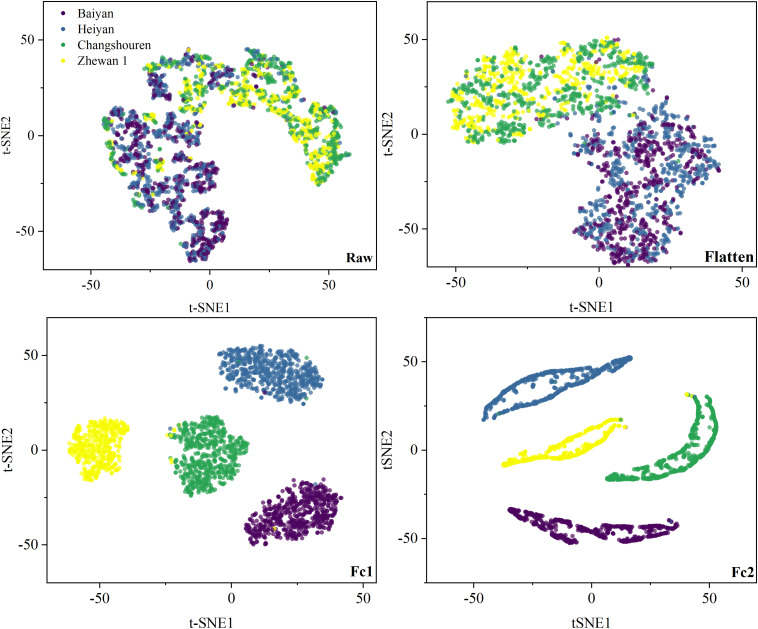
Feature visualization of VGG-MODEL on the Pea dataset, using t-SNE.

Visualizing the feature distribution at each layer of the deep network was an important channel to understand the training process of the deep model (Lin and Maji, 2016; Zintgraf et al., 2017; Zhang and Zhu, 2018). In this study, the t-SNE technique was used to visualize the original high-dimensional spectra and the features output by the flatten, Fc1, and Fc2 layers of the deep model in a two-dimensional space, as shown in Figure 6 and Supplementary Figures 1–8. For the Pea dataset, the raw spectral samples were aggregated into two categories, consistent with the average spectral analysis. After passing the flatten layer, the spectra with easily confused categories like Baiyan and Heiyan, or Changshouren and Zhewan 1, gradually became distinguishable. As the layers deepened, the samples within a category were clustered closely, while those between different categories became discrete. The samples were clearly gathered into four categories after output by the Fc2 layer. It could be seen that the deep model gradually transformed the samples from a cluttered state to a distinguishable state. It was why the deep model could obtain better performance than the traditional methods.

For the four target datasets, the raw spectra in the Rice and Oat datasets, especially in the Rice dataset, were slightly more regular than those in the Wheat and Cotton datasets. The variety Yongyou9 was strongly distinguishable from the other two varieties. This phenomenon was also consistent with previous spectral analysis. Thus, most traditional methods performed better on the Rice and Oat datasets than on the other two datasets. Since all the weights before the flatten layer were transferred from the deep model based on the source Pea dataset or the Indian-Pines dataset directly, the features output by the flatten layer of the deep transferred model contained the spectral patterns learned from these two datasets. From Supplementary Figures 1–4, it could be seen that, for the Rice, Oat, and Cotton datasets, the features output by the flatten layer presented a more aggregated distribution pattern than the raw spectral samples. In Supplementary Figures 5–8, however, the distribution patterns of the features output by the flatten layer were not significantly improved compared with the raw spectral samples. These results intuitively illustrated the critical role of the shared features for transfer learning. Effective transfer learning was conductive to the classification of different varieties of seeds in this study. The spectral features learned from the source Pea dataset were reused through transferring, facilitating the classification of small target datasets. The Wheat dataset might be too cluttered so that the output of the flatten layer did not show distinguishability. The target datasets began to work from the Fc1 layer. The samples gradually showed strong separability with the layers deepened. After output by the final Fc2 layer, the rice and oat samples had been divided into three and four categories, respectively. Thus, the deep transferred model achieved two high accuracies of 95 and 99%. However, the wheat and the cotton samples still had some overlapping phenomenon, which led to relatively low accuracies of the deep transferred model. Since there were no effective features transferred, the features output by the Fc1 and Fc2 layer in Supplementary Figures 5–8 showed a more discrete distribution pattern than those in Supplementary Figures 1–4. This was why the classification performance of the transfer model based on the Indian-Pines dataset was worse than that based on the source Pea dataset.

**FIGURE 7:**
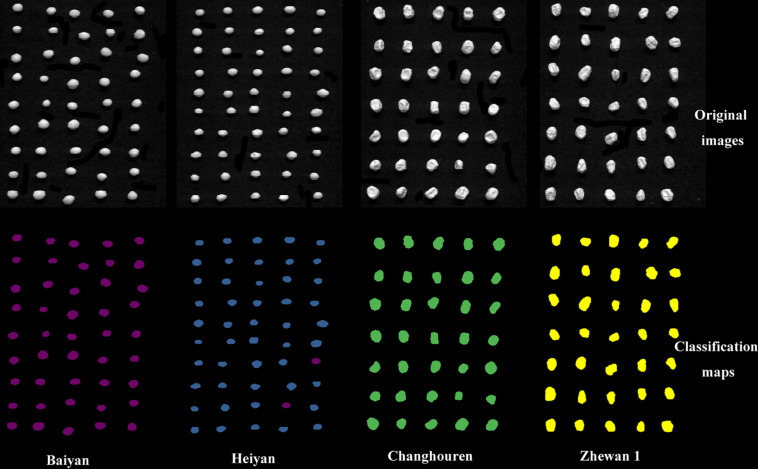
The classification visual maps of pea seeds.

The classification visualization of crop seeds was helpful for breeders to select varieties that meet requirements and for market supervision authorities to check seed purity. In this study, the categories of pea seeds classified by the optimal model, VGG-MODEL, were visualized in a map. As shown in Figure 7, Baiyan and Heiyan showed similar smooth texture features in the original hyperspectral images. In contrast, Changshouren and Zhewan 1 showed rough texture due to water loss during the drying process. According to human vision, these four varieties were naturally divided into two categories, consistent with the visualization analysis of the distribution of the samples. Among the predicted 180 seeds, only two seeds of the variety Heiyan were misclassified into the similar Baiyan category. This accuracy was sufficient for variety selection during the breeding process or purity detection in actual production. The characteristics of batch detection of HSI combined with the capabilities of rapid analysis of deep transfer learning may provide a brand-new solution for identifying crop varieties under sample-limited condition. It is expected to help accelerate the process of crop variety screening.

## Conclusion

This study attempted to use HSI and deep transfer learning to achieve accurate and rapid varieties classification of crop seeds under sample-limited condition. The VGG-MODEL based on the sample-rich dataset stood out from three deep neural networks with typical structures and was utilized as the deep source model to be transferred. The transfer results on the four small target datasets showed that the deep transferred model could fully use the shared spectral features of crop seeds extracted by the source deep model. The deep transferred model could achieve better performance than traditional multivariate analysis methods under sample-limited condition, especially when using tiny samples. Giving a glimpse into the process of deep transfer learning, the visualization of the feature distribution at each layer of the deep network further confirmed the portability of shared spectral features. It revealed why the deep network achieved high accuracy. The visualization of classification results provided an intuitive and convenient manner for varieties classification of crop seeds. In conclusion, HSI combined with deep transfer learning, was a great potential tool for the classification of seed varieties with limited samples, which will significantly accelerate the seed screening process in fields with scarce samples. This study also provided a new idea for detecting other qualities of crop seeds based on HSI under sample-limited condition.

## Data Availability Statement

The raw data supporting the conclusions of this article will be made available by the authors, without undue reservation.

## Author Contributions

NW, FL, and CZ conceived the research concept. NW and CZ performed the experiments. NW wrote the manuscript. FL, FM, ML, and YH contributed to the results analysis and discussion. YH provided financial support. All the authors contributed to the study and approved the submitted version.

## Conflict of Interest

The authors declare that the research was conducted in the absence of any commercial or financial relationships that could be construed as a potential conflict of interest.

## Publisher’s Note

All claims expressed in this article are solely those of the authors and do not necessarily represent those of their affiliated organizations, or those of the publisher, the editors and the reviewers. Any product that may be evaluated in this article, or claim that may be made by its manufacturer, is not guaranteed or endorsed by the publisher.

## Footnotes

1 http://www.ehu.eus/ccwintco/index.php?title=Hyperspectral_Remote_Sensing_Scenes

2 https://scikit-learn.org/stable/
