# Supplementary material for: Rapid and Accurate Varieties Classification of Different Crop Seeds Under Sample-Limited Condition Based on Hyperspectral Imaging and Deep Transfer Learning
Source: Front Bioeng Biotechnol. 2021 Jul 23;9:696292. doi: 10.3389/fbioe.2021.696292 (PMC8343196; doi:10.3389/fbioe.2021.696292)
Supplement: Supplementary file 1 [file Table_1.DOCX]

Supplementary Material

# Supplementary Material 1

**Table S1** The detailed parameters of three deep neural networks

| **Models** | | **#Filters**^1^ | **Kernel_size** | **Strides** | **Padding** | **Pool_size** |
| --- | --- | --- | --- | --- | --- | --- |
| **VGG-MODEL** | **V block1** | 16 | 3 | 1 | same | - |
|  | **V block2** | 32 | 3 | 1 | same | - |
|  | **Max pooling** | - | - | 2 | same | 2 |
| **RES-MODEL** | **Conv-1D** | 32 | 7 | 2 | valid | - |
|  | **Max pooling** | - | - | 2 | same | 3 |
|  | **R block1** | 32 | 3 | 1 | same | - |
|  | **R block2** | 64 | 3 | 2 | same | - |
|  | **R block3** | 64 | 3 | 1 | same | - |
|  | **R block4** | 128 | 3 | 2 | same | - |
| **INCEPTION-MODEL** | **Conv-1D** | 16 | 3 | 2 | valid | - |
|  | **Max pooling** | - | - | 2 | same | 3 |
|  | **I block1** | (8), (8, 16), (4, 4), (4) | - | 1 | same | - |
|  | **I block2** | (16), (16, 32), (4, 8), (8) | - | 1 | same | - |
|  | **I block3** | (32), (32, 64), (8, 16), (16) | - | 1 | same | - |
|  | **I block4** | (64), (64, 128), (16, 32), (32) | - | 1 | same | - |

Note: the #Filters^1^ parameter of V block and R block represented the number of convolution filters. the #Filters parameter of I block represented the number of convolution filters in the four branches of this module.

# Supplementary Material 2

**Supplementary Figures Captions**

Figure S1. Feature visualization of VGG-MODEL on the Rice dataset using t-SNE.

Figure S2. Feature visualization of VGG-MODEL on the Oat dataset using t-SNE.

Figure S3. Feature visualization of VGG-MODEL on the Wheat dataset using t-SNE.

Figure S4. Feature visualization of VGG-MODEL on the Cotton dataset using t-SNE.

Figure S5. Feature visualization of MODEL0 on the Rice dataset using t-SNE.

Figure S6. Feature visualization of MODEL0 on the Oat dataset using t-SNE.

Figure S7. Feature visualization of MODEL0 on the Wheat dataset using t-SNE.

Figure S8. Feature visualization of MODEL0 on the Cotton dataset using t-SNE.
