# Supplementary figures and images for: Rapid and Accurate Varieties Classification of Different Crop Seeds Under Sample-Limited Condition Based on Hyperspectral Imaging and Deep Transfer Learning
Source: Front Bioeng Biotechnol. 2021 Jul 23;9:696292. doi: 10.3389/fbioe.2021.696292 (PMC8343196; doi:10.3389/fbioe.2021.696292)

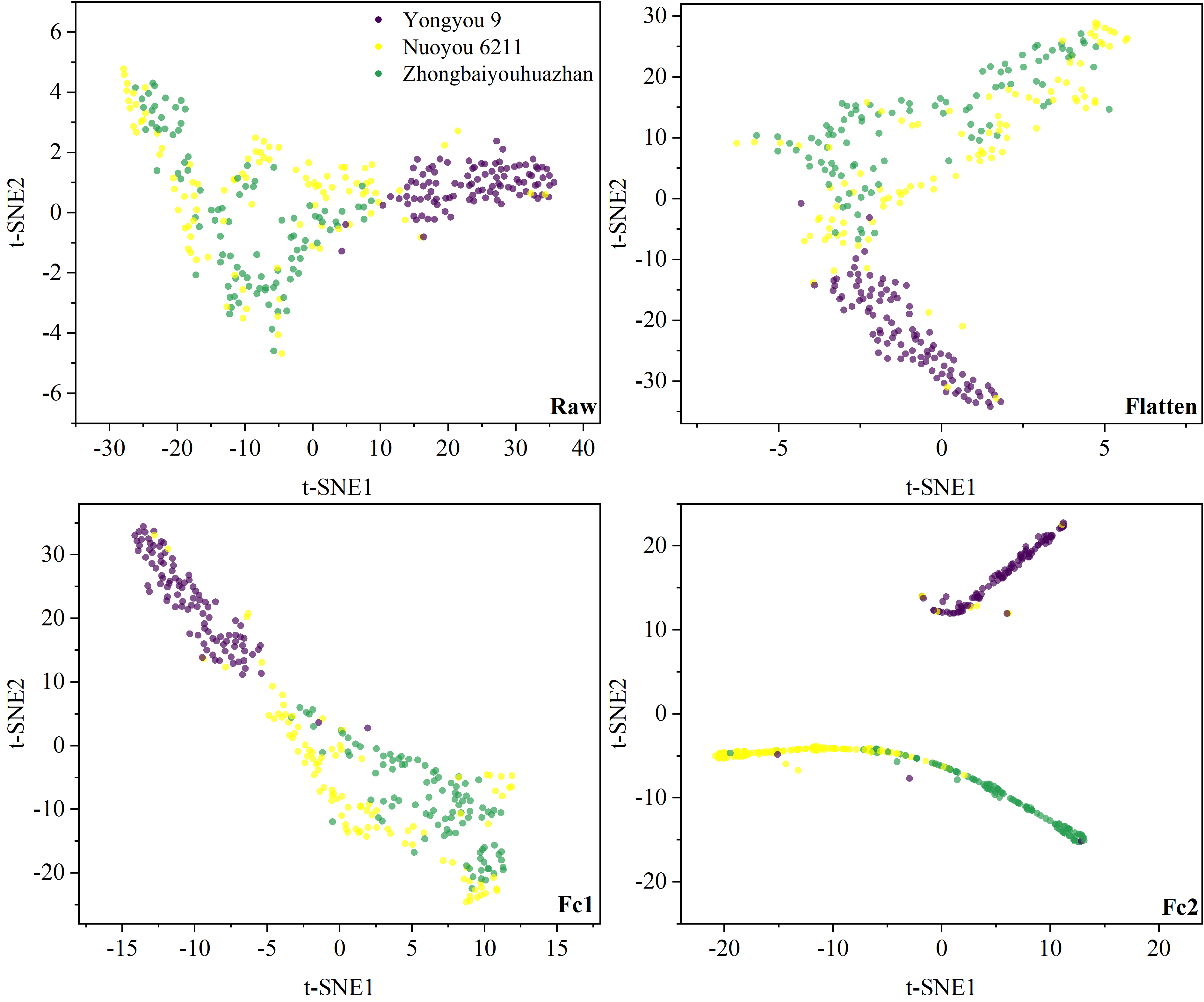

Supplement: Supplementary file 6 [file Image_5.TIF]

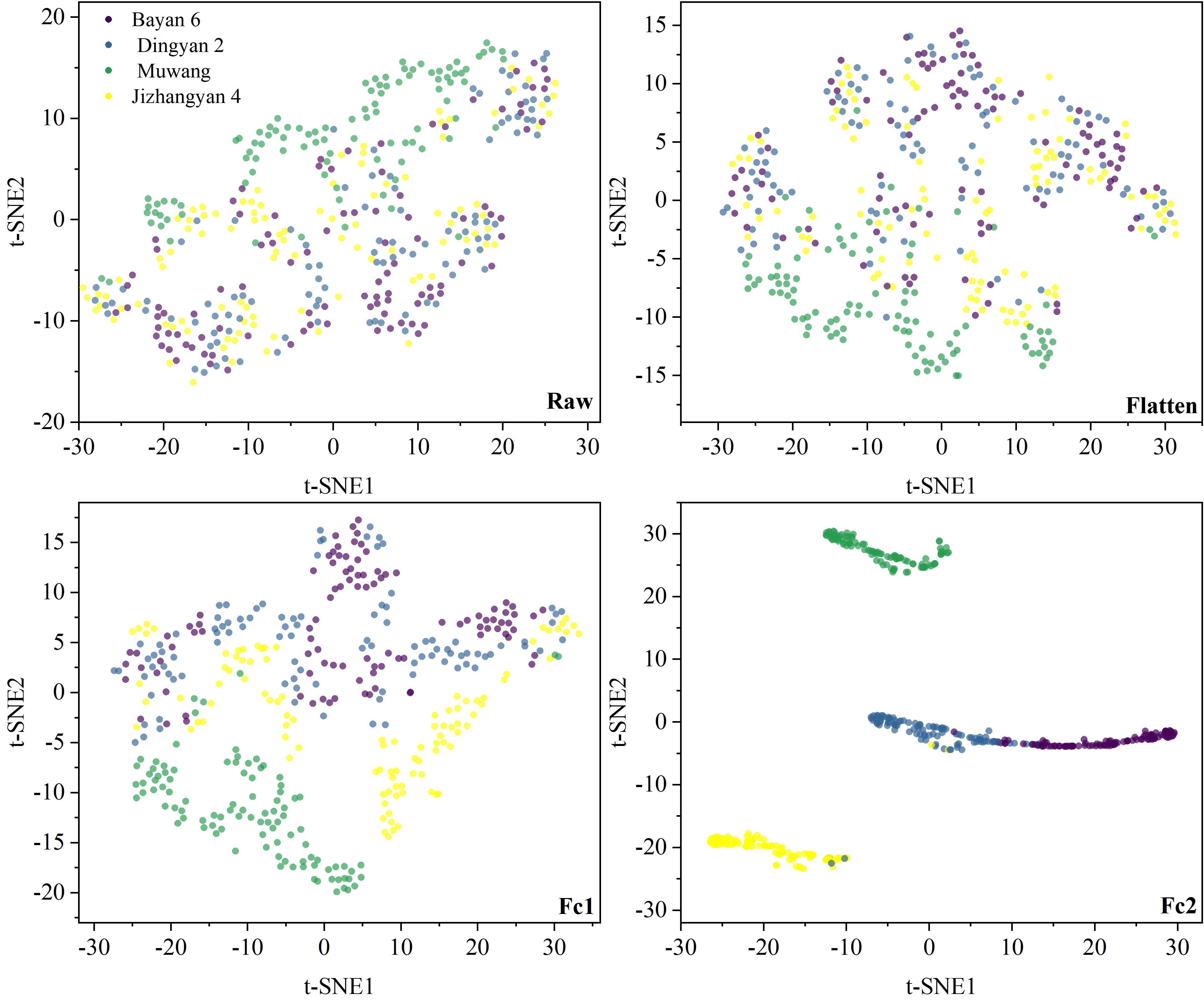

Supplement: Supplementary file 7 [file Image_6.TIF]

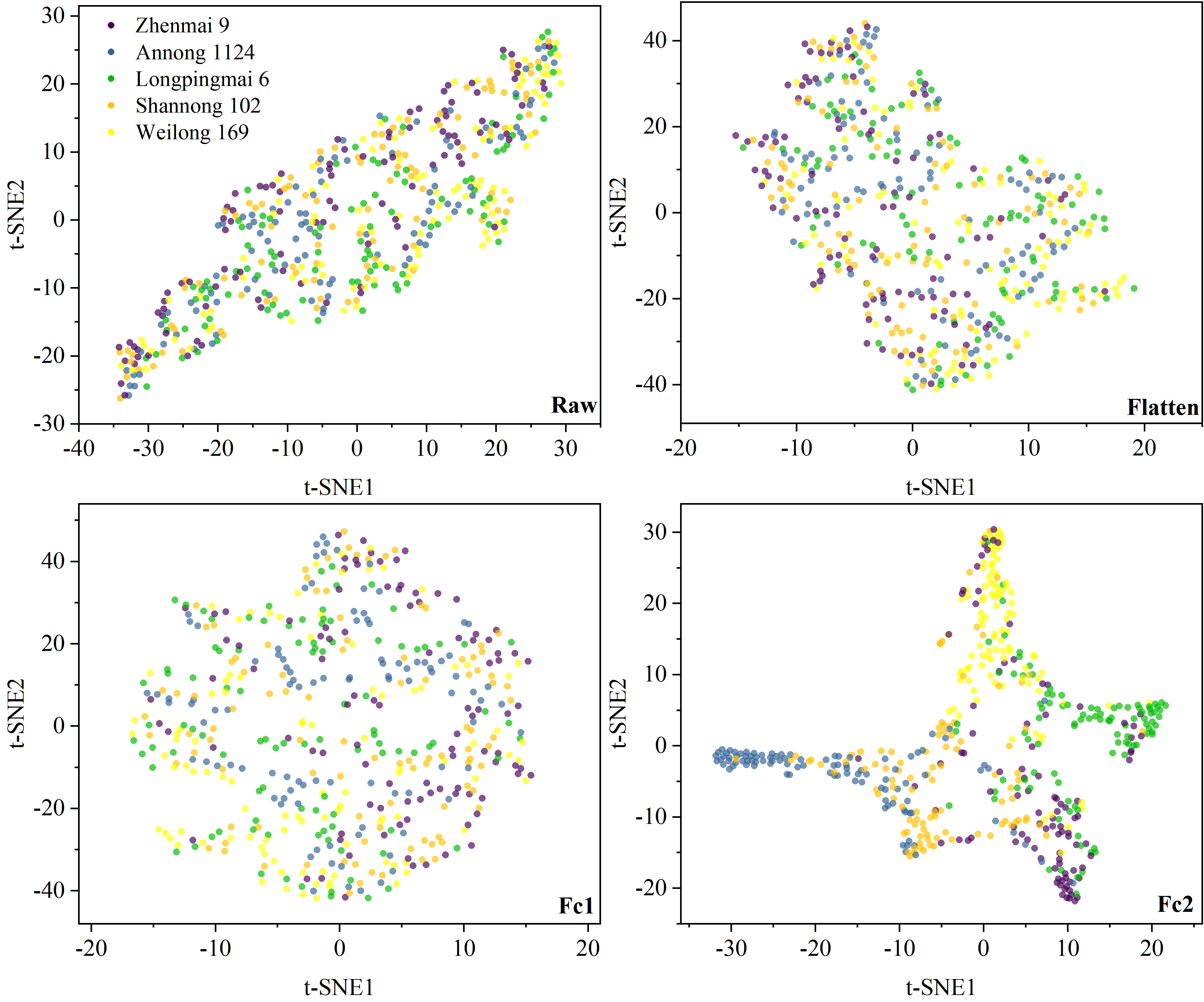

Supplement: Supplementary file 8 [file Image_7.TIF]

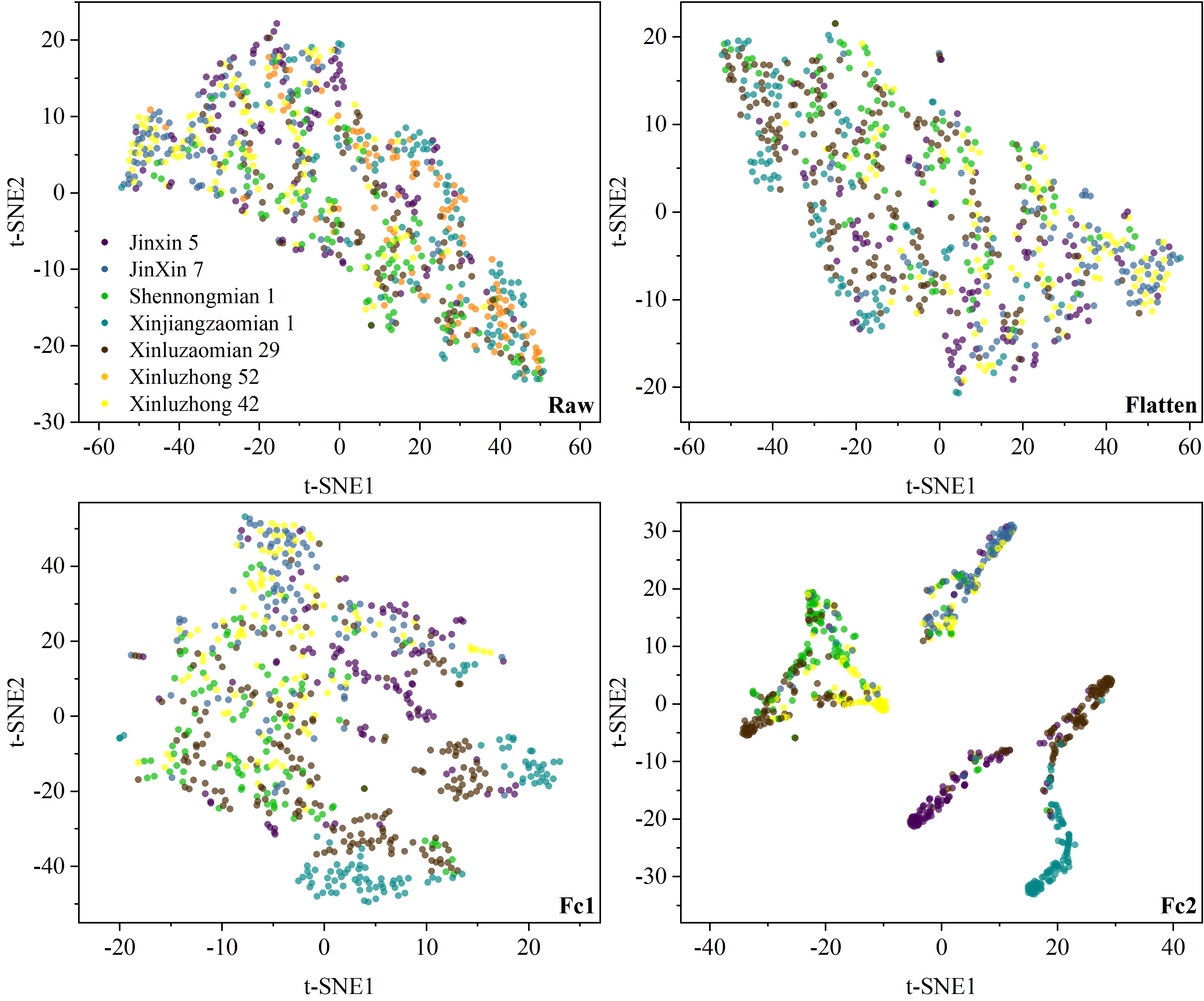

Supplement: Supplementary file 9 [file Image_8.TIF]
